# Bisphosphonates as treatment of secondary osteoporosis in children: a case series

**DOI:** 10.1186/1687-9856-2015-S1-P67

**Published:** 2015-04-28

**Authors:** Frida Soesanti, Fitria Mayasari, Aman B Pulungan

**Affiliations:** 1 Pediatric Endocrinology Division, Faculty of Medicine, University of Indonesia-Cipto Mangunkusumo hospital, Jakarta, Indonesia; 2 Pediatrics Departement, University of Indonesia, Jakarta, Indonesia

**Keywords:** secondary osteoporosis, bone mineral density, bisphosphonates

## Background

Secondary osteoporosis due to chronic disease is a major pediatric health concern and has certain unique diagnostic with various clinical challenges. Bisphosphonates has been used in small numbers of pediatric patients to treat secondary osteoporosis resulted in significantly fewer fractures and improved mobility.

## Objective

To present case series of secondary osteoporosis due to chronic disease treated with biphosphonate.

## Case Series

We included 6 patients (2 boys, 4 girls), 7–15 years of age with secondary osteoporosis who were treated with biphosphonate. Two patients were diagnosed acute lymphoblastic leukemia, two patients with systemic lupus erythematosus and others with juvenile rheumatoid arthritis. Two patients received methotrexate and dexamethasone; one patient was treated with methotrexate and prednisone; two patients with methylprednisolone; and rest with only methotrexate. Four of them suffered back pain due to trauma and from radiographic examination showed multiple compression fracture on the vertebrae. Lumbal BMD Z-score was ranging from -2.8 to -5.1 in patients who had fracture, while in patients without fracture -2.6 to -3.1. We used zoledronic acid 0.05 mg/kgBW. Two patients already received pamidronate as previous treatment. In 6 - 12 months of follow-up, there were evidence of reduced pain, improved mobility and BMD Z-score, also no evidence of new fractures in radiographic evaluation.

## Conclusion

Bisphosphonates appears to be safe and effective as treatment for children with secondary osteoporosis.

